# Travel Characteristics Analysis and Traffic Prediction Modeling Based on Online Car-Hailing Operational Data Sets

**DOI:** 10.3390/e23101305

**Published:** 2021-10-04

**Authors:** Shenghan Zhou, Bang Chen, Houxiang Liu, Xinpeng Ji, Chaofan Wei, Wenbing Chang, Yiyong Xiao

**Affiliations:** School of Reliability and Systems Engineering, Beihang University, Beijing 100191, China; zhoush@buaa.edu.cn (S.Z.); bang@buaa.edu.cn (B.C.); zy1914125@buaa.edu.cn (H.L.); sy1914103@buaa.edu.cn (X.J.); zy2014214@buaa.edu.cn (C.W.); changwenbing@buaa.edu.cn (W.C.)

**Keywords:** online car-hailing, travel characteristics analysis, traffic prediction modeling, multivariate variables time series, hybrid model

## Abstract

Smart transportation is an important part of smart urban areas, and travel characteristics analysis and traffic prediction modeling are the two key technical measures of building smart transportation systems. Although online car-hailing has developed rapidly and has a large number of users, most of the studies on travel characteristics do not focus on online car-hailing, but instead on taxis, buses, metros, and other traditional means of transportation. The traditional univariate variable hybrid time series traffic prediction model based on the autoregressive integrated moving average (ARIMA) ignores other explanatory variables. To fill the research gap on online car-hailing travel characteristics analysis and overcome the shortcomings of the univariate variable hybrid time series traffic prediction model based on ARIMA, based on online car-hailing operational data sets, we analyzed the online car-hailing travel characteristics from multiple dimensions, such as district, time, traffic jams, weather, air quality, and temperature. A traffic prediction method suitable for multivariate variables hybrid time series modeling is proposed in this paper, which uses the maximal information coefficient (MIC) to perform feature selection, and fuses autoregressive integrated moving average with explanatory variable (ARIMAX) and long short-term memory (LSTM) for data regression. The effectiveness of the proposed multivariate variables hybrid time series traffic prediction model was verified on the online car-hailing operational data sets.

## 1. Introduction

In the 21st century, the rapid development and wide application of modern information communication technologies, such as the Internet of Things (IoT), cloud computing, big data, and mobile Internet, have led to changes in urban development [1,2]. Therefore, smart urban cities have rapidly become a research hotspot, as a concept devoted to using modern information communication technologies to realize the smart management of urban areas, create a better life for urban areas’ users and residents, and promote the sustainable development of urban areas [3]. Urban traffic is common, and smart transportation is an important part of smart urban construction [4]. Smart transportation involves the effective optimization of traffic operation management and provides intelligent service for vehicles and travelers, which can create a green and safe travel environment for the public and improve quality of life [5,6]. Traffic big data are the basis of intelligent transportation [7]. With the help of data mining, we can mine the travel characteristics from historical traffic big data; with the help of machine learning, we can use historical traffic big data to train the prediction model. Understanding travel characteristics and having prediction models can provide a powerful decision-making basis for traffic management and vehicle scheduling.

Traffic and travel characteristics studies aim to determine the traffic behavior of people living in urban areas, and the relationship between this behavior and the objective environment (such as the social environment, urban environment, natural geographical environment, etc.) [8]. The research purpose is to discover the patterns of behavior, evolution law, and reaction mechanism of urban traffic using traffic big data, and applying the results to the planning, design, construction, and management of urban transportation. Xiao et al. [9] mined the travel history data from the Capital Bikeshare system in the Washington, DC area to study the travel characteristics of shared-bikes users from the aspects of travel demand, travel flow, and so on. Li et al. [10] analyzed the spatial and temporal travel characteristics of residents’ travel by e-bike based on real-time global positioning system (GPS) data in the central area of Tengzhou City, Shandong Province, China. Wang et al. [11] conducted a large-scale study using taxi GPS data collected from more than 25,000 drivers for seven consecutive days in Beijing, China, to reveal the spatial-temporal characteristics of residents’ travel by taxi. Yu et al. [12] used a heat map to study the spatial–temporal characteristics of bus travel demand based on smart card data and bus GPS data provided by the Guangzhou transit agency. Goel et al. [13] studied the characteristics of metro travel by conducting an on-board survey of 1112 Delhi metro passengers in 2011. In summary, scholars have conducted detailed studies on the travel characteristics of the urban people under different modes of transportation, including shared-bikes, e-bikes, taxis, buses, and metros. However, few studies have been conducted on the characteristics of online car-hailing. As a typical product driven by the sharing economy, online car-hailing has attracted a large number of users because of its convenience, speed, flexibility, and lower price, and has become an important means of transportation for urban people [14,15]. Therefore, it is necessary to study the travel characteristics of online car-hailing.

The research object of traffic prediction model is usually related to variables or states of urban traffic, such as traffic flow [16], traffic demand [17], traffic speed [18], and traffic jams [19]. These variables or states change often with time, so time series models are often used to predict traffic. According to different assumptions, time series models can be divided into linear time series models and nonlinear time series models [20]. The linear time series models, such as autoregressive moving average (ARMA), ARIMA, and ARIMAX, are widely used in the field of early traffic prediction. Klepsch et al. [21] proposed an approximating vector model based on ARMA and principal component analysis (PCA) to predict the traffic speed of highways. Xu et al. [22] developed a real-time road traffic state prediction model based on ARIMA and the Kalman filter, which provides improved prediction accuracy. Williams [23] applied ARIMAX for short-term highway traffic flow prediction and verified the effectiveness of the proposed model on a real dataset from France. Linear time series models are simple and can fit the linear time series well, but they are ineffective in dealing with nonlinear time series [24]. Therefore, many nonlinear time series models based on machine learning have been proposed for traffic prediction. Hu et al. [25] proposed a short-term traffic flow prediction model based on support vector regression (SVR) and used particle swarm optimization (PSO) to search for the optimal SVR parameters, providing higher precision with less learning time. Alajali et al. [26] developed an ensemble decision tree model based on gradient boosting regression tree (GBRT), random forest (RF), and extreme gradient boosting (XGBoost) for traffic flow prediction at intersections. Tian et al. [27] presented a new approach for learning the traffic flow prediction residuals by explicitly combining the missing patterns based on the revised LSTM model, which overcomes the problem of missing data. Nonlinear time series models can fit the nonlinear time series well, but they are not effective in dealing with linear time series [28]. Therefore, some hybrid time series models have been proposed. Zhang et al. [29] constructed a novel hybrid methodology by combining ARIMA and SVR to predict traffic flow on highways and proved the superiority of hybrid model compared with the single model. Liu et al. [30] reported a traffic flow combination forecasting method based on ARIMA and LSTM and designed an adaptive traffic flow embedded system. Although these hybrid models based on ARIMA achieved good results, ARIMA is only suitable for univariate time series modeling [31], which means all these univariate forecast models do not consider district, weather, or other variables, but these variables impact traffic flow and demand. Therefore, if they can be considered in the prediction model, the performance of the model will be improved.

Therefore, to fill the gap in research on the characteristics of online car-hailing and to overcome the shortcomings of univariate hybrid time series models in traffic prediction, we aimed to analyze travel characteristics and conduct traffic demand prediction modeling based on online car-hailing operational data sets. Specifically, based on the online car-hailing operational data sets, we investigated online car-hailing travel characteristics from the aspects of district, time, traffic jams, weather, air quality, and temperature. The analysis of online car-hailing travel characteristics from the perspective of these parameters will help online car-hailing drivers, passengers, and platforms, and traffic management departments to capture the changes in online car-hailing travel when these parameters or the external environment change. For example, understanding online car-hailing travel district characteristics can help online car-hailing drivers to find hotspot help online car-hailing platforms to find taxi hotspot times to generate a targeted order distribution strategy; understanding travel traffic jam characteristics analysis can help traffic management departments to identify how the traffic jams change with traffic demand; and understanding online car-hailing travel weather type, temperature, and air quality characteristics analysis can help the passengers to choose reasonable transport and create a reasonable travel strategy. On the basis of the mined online car-hailing travel characteristics, in this paper, a traffic prediction method suitable for multivariate variables time series modeling is proposed to overcome the limitations of the single model, which uses MIC for feature selection, and fuses ARIMAX and LSTM to perform data regression. The proposed online car-hailing demand prediction model will also play various significant roles. From the perspective of online car-hailing drivers, the demand prediction model can help them know the demand in different districts in advance, so that they receive more orders. From the perspective of online car-hailing passengers, the demand prediction model can help them know when and where the demand is low, so that they can easily order a car. From the perspective of online car-hailing platforms, they can use the predicted results to reasonably dispatch cars in different areas to improve the overall operation efficiency. From the perspective of traffic management departments, the predicted results can be used to guide the management of roads and vehicles.

The main objectives of this study are as follows:

(1) Study the online car-hailing travel characteristics from multiple dimensions to support the traffic management and traffic prediction modeling.

(2) Develop a traffic prediction method suitable for multivariate variables hybrid time series modeling to introduce the explanatory variables to improve the performance of time series models.

The main contributions of this study are as follows:

(1) A set of analyses and a processing framework for online car-hailing data sets is proposed for online car-hailing travel characteristics analysis and prediction modeling.

(2) An analysis is provided of the online car-hailing travel characteristics from the aspects of district, time, traffic jams, weather, air quality, and temperature based on the online car-hailing operational data sets.

(3) A novel traffic prediction method suitable for multivariate variables hybrid time series modeling is proposed based on MIC, ARIMAX, and LSTM.

The remaining part of this paper is organized as follows: In Section 2, the basic information of the online car-hailing operational data sets and the data preprocessing operations are introduced. Section 3 describes the online car-hailing travel characteristics in multiple dimensions based on the considered data sets. Section 4 introduces the basic principle of the multivariate hybrid time series model, and verifies the proposed model on the online car-hailing operational data sets. Section 5 describes the conclusions and Section 6 provides a discussion.

## 2. Data Overview and Preprocessing

### 2.1. Data Overview

The data sets used in this study were obtained from the Didi Chuxing GAIA Initiative, which recorded the Didi online car-hailing operational information in Hangzhou from 1–21 January 2016. The Didi Chuxing GAIA Initiative used the grid method to divide Hangzhou into 66 non-overlapping small square districts. According to the divided districts, the order information was recorded to obtain the order data set, and the traffic jam information was recorded to obtain the traffic jam data set. In addition, as the online car-Hailing demand is often affected by the weather conditions, the weather information of Hangzhou was also recorded every five minutes to obtain the weather data set. These data sets provide order, traffic jam, and the weather information, and are helpful for analyzing and studying the online car-hailing travel characteristics in Hangzhou from multiple dimensions.

The order data set contains all the orders that occurred in Hangzhou from 1 to 21 January 2016, approximately 8.5 million data records. The order data set contain six fields: order_id, driver_id, passenger_id, start_district_id, dest_district_id, and datetime. The description and examples of each field are shown in Table 1. Among them, driver_id may be null, which means this order had no driver response so this online car-hailing demand was not met.

The traffic jam data set provides the traffic jam states every ten minutes in the defined 66 districts from 1–21 January 2016. The traffic jam data set contains about 920,000 data records and covers three fields: district_id, traffic, and datetime. The description and examples of each field are shown in Table 2. Among them, traffic field is composed of traffic jam level and road quantity, and different traffic jam levels are separated by spaces. For example, “1:231” means that there are 231 roads in traffic jam level 1; the larger the traffic jam level, the worse the jam.

The weather data set contains the weather information of Hangzhou every five minutes from 1–21 January 2016, for a total of about 6000 data records. The weather data set covers four fields: datetime, weather, temperature, and air_quality. The description and examples of each field are provided in Table 3. Among them, the weather type code is: 1, cloudy; 2, overcast; 3, shower; 4, thundershower; 8, moderate rain; and 9, heavy rain; the higher the air quality level, the worse the air quality.

### 2.2. Data Preprocessing

**Figure 1:**
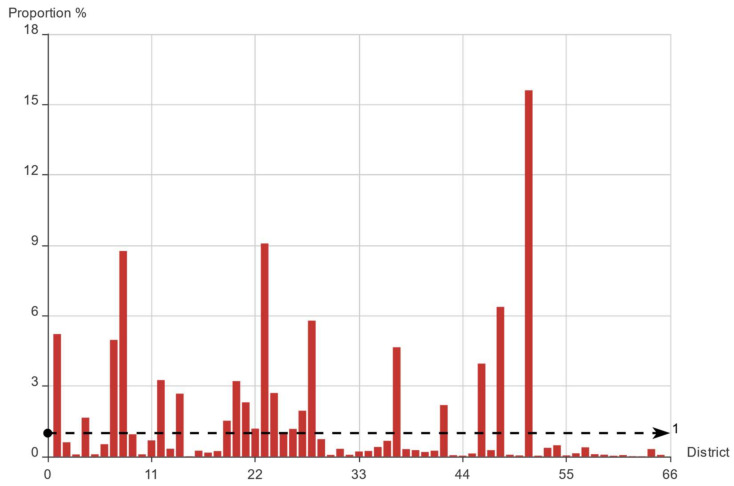
Average daily online car-hailing demand proportion in every district.

The original data sets cannot be used directly, so a series of data preprocessing operations must be applied. We firstly counted the average daily online car-hailing demand proportion in every district, and the specific results are shown in Figure 1. From Figure 1, the online car-hailing demand in Hangzhou from 1 to 21 January 2016 the among 66 districts shows an obvious Pareto principle [32], which means unbalanced distribution, such as 20% of the population owning 80% of the wealth. Specifically, 45 districts had less than 1% of the total daily online car-hailing demand, whereas the average daily online car-hailing demand of the remaining 21 districts accounted for more than 89% total daily demand. The data sets of the districts with low demand often show large data fluctuations, which will affect the online car-hailing travel characteristics analysis and prediction modeling.

Therefore, in this study, the data sets of the above 45 districts with lower demand were first deleted, and only the data sets of the 21 districts with higher demand were retained. Then, according to the characteristics of the data sets and the actual situation, the 24 h of a day from 0:00:00 to 23:59:59 were divided into 144 time slices, every ten minutes, which were coded from 1 to 144. To study the online car-hailing travel characteristics, we defined five variables based on the order data set:

(1) Demand D: the number of records with the unique order ID in the order data set in the current time slice of the current district;

(2) Demand unmet $D_{u}$: the number of records with a null driver ID in the order data set in the current time slice of the current district;

(3) Demand met $D_{m}$: the difference between demand D and demand unmet $D_{u}$;

(4) Passenger inflow $P_{i}$: the number of records whose driver ID is not null and the order destination district is the current district in the order data set in the current time slice;

(5) Passenger outflow $P_{o}$: the number of records whose driver ID is not null and the order start district is the current district in the order data set in the current time slice.

Finally, after a series of preprocessing operations, such as filling missing data and coding discrete variables, about 60,000 data records were obtained. Taking district 16 as an example, some of its combined demand data are shown in Table 4.

## 3. Online Car-Hailing Travel Characteristics Analysis

As an important means of transportation for urban people, online car-hailing often presents various characteristics due to the influence of many factors, so these characteristics must be explored for traffic management purposes. Therefore, based on considered data sets, we mined the online car-hailing travel characteristics from multiple dimensions such as the district, time, traffic jams, weather, air quality, and temperature.

### 3.1. Online Car-Hailing Travel: District Characteristics Analysis

**Figure 2:**
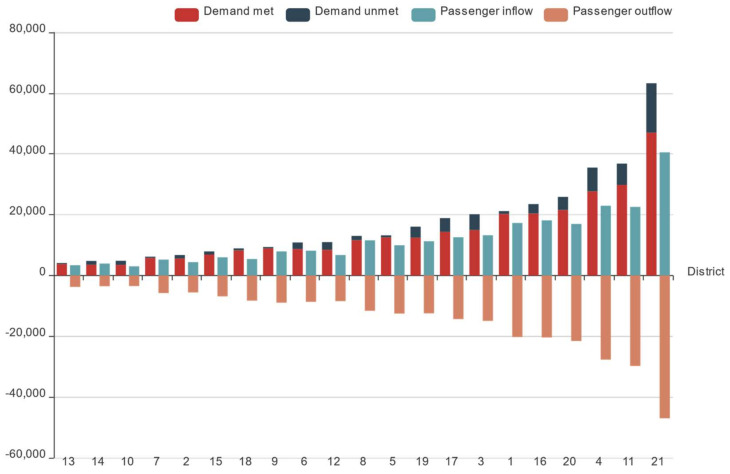
Online car-hailing travel differences in the different districts.

To reflect the differences in online car-hailing among districts as a whole, the average values of demand D, demand unmet $D_{u}$, demand met $D_{m}$, passenger inflow $P_{i}$, and passenger outflow $P_{o}$ from 1 to 21 January 2016 were counted and the specific results are shown in Figure 2. From Figure 2, the online car-hailing in Hangzhou presents obvious district characteristics. The demand in districts 3, 1, 16, 20, 4, 11, and 21 was relatively high, and the daily average demand were all more than 20,000. However, the demand doe online car-hailing in districts 13, 14, 10, 7, and 2 was relatively low, with a daily average demand of less than 7000. In these districts with higher demand, the passenger inflow and outflow were more frequent, but the unmet demand $D_{u}$ was also higher. We found that passenger outflow $P_{o}$ was greater than passenger inflow $P_{i}$ in most districts, which indicates that most of the citizens in Hangzhou preferred to use online car-hailing for their departure trip.

### 3.2. Online Car-Hailing Travel: Time Characteristics Analysis

**Figure 3:**
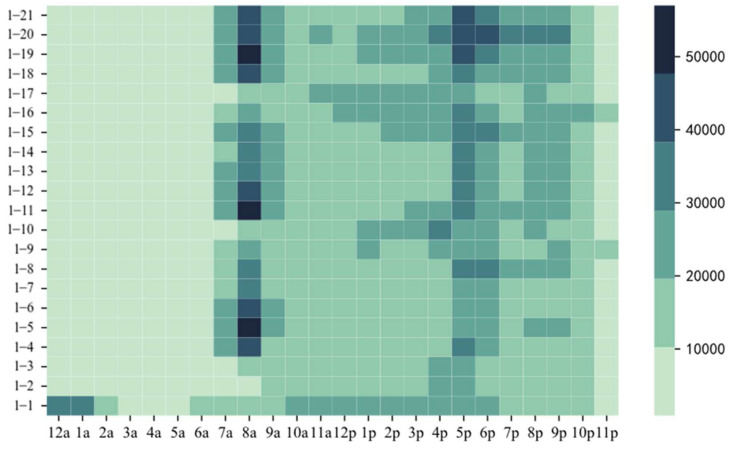
Online car-hailing demand time heat map.

Human activities have obvious time characteristics. Online car-hailing, as an important means of transportation, also has obvious time characteristics. Therefore, we obtained the statistics of the 24 h demand of online car-hailing in Hangzhou from 1 to 21 January 2016, and obtained the demand time heat map, as shown in Figure 3. Figure 3 shows that the daily online car-hailing demand in Hangzhou had obvious double hump feature, that is, during the morning rush and the evening rush. Specifically, the morning rush occurred from 7:00 to 9:00 a.m. and the evening rush from 4:00 to 6:00 p.m. Moreover, the online car-hailing demand was maintained at a low level starting from 11:00 p.m. However, an abnormal rush occurred after 12:00 p.m. on January 1, which may be caused by New Year’s Day. It can be seen that people’s travel characteristics differed on different dates.

**Figure 4:**
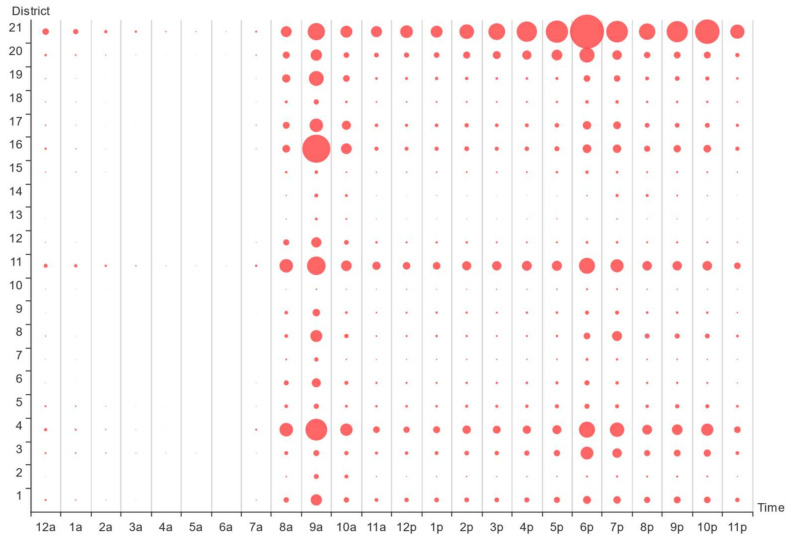
Time bubble map of online car-hailing demand on workdays.

To study the online car-hailing travel characteristics on workdays, we counted the average 24 h demand in 21 districts on work days, and obtained the time bubble map of online car-hailing demand on work day, as shown in Figure 4. From Figure 4, Hangzhou citizens’ demand for online car-hailing on workdays shows a more obvious double hump feature: the demand in district 21 during the morning rush was significantly lower than during in the evening rush, whereas the demand in districts 4, 11, 16, and 19 during the morning peak was significantly higher than during the evening peak. Therefore, we speculated that district 21 may belong to a typical working district, whereas districts 4, 11, 16, and 19 may be typical residential districts.

**Figure 5:**
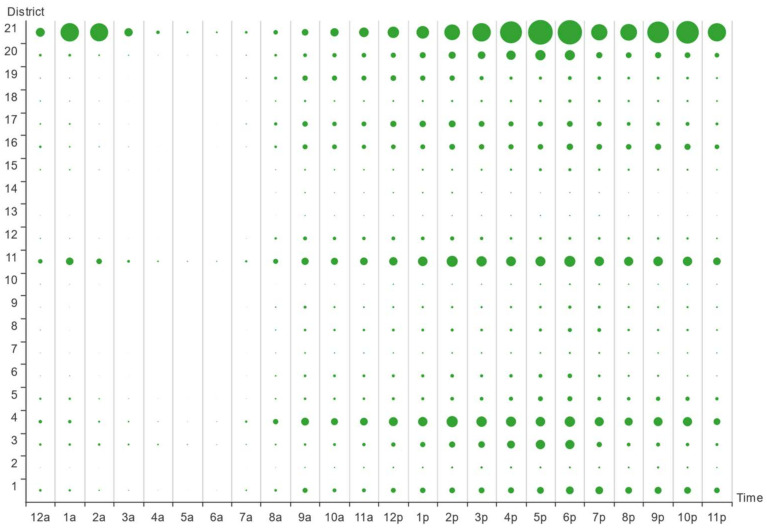
Time bubble map of online car-hailing demand on nonworking days.

To study the online car-hailing travel characteristics on nonwork days, we counted the average 24 h demand in 21 districts on these days, and obtained the time bubble map of online car-hailing demand on nonworking days, as shown in Figure 5. From Figure 5, Hangzhou citizens’ demand for online car-hailing on nonworking days shows a certain single hump feature, with only an evening peak. Moreover, the demand on nonworking days is generally lower, indicating that Hangzhou citizens’ travel desire is lower on nonworking days, which may be caused by the winter season and lower temperatures in January.

### 3.3. Online Car-Hailing Travel: Traffic Jam Characteristics Analysis

**Figure 6:**
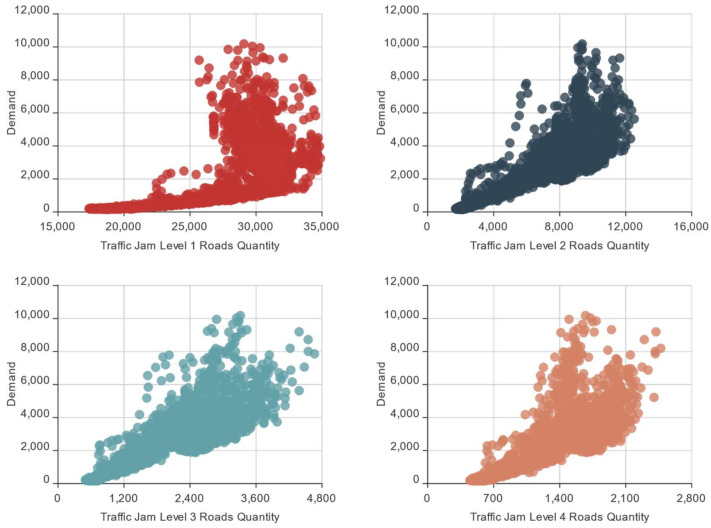
Online car-hailing demand and traffic jam scatter map.

Traffic jams also affect people’s selection of transportation, and vice versa. Therefore, to study the online car-hailing travel traffic jam characteristics, we counted the online car-hailing demand and the road quantities in different traffic jam levels in every time slice to obtain the online car-hailing demand and traffic jam scatter map, as shown in Figure 6. From Figure 6, the online car-hailing demand increased with traffic jams, showing an obvious positive correlation. The higher the traffic jam level, the more slowly the road quantities grew with the online car-hailing demand, which conforms to the general rule of traffic jam spread.

### 3.4. Online Car-Hailing Travel: Other Characteristics Analysis

**Figure 7:**
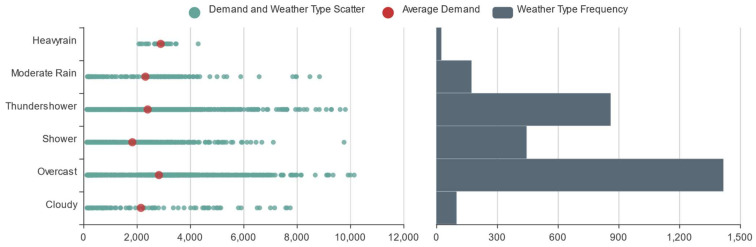
Online car-hailing demand and weather type scatter frequency map.

Many studies have shown that weather type [33], temperature [34], and air quality [35], as external environmental factors, also have a direct or indirect impact on travel characteristics. To study how the weather characteristics affected online car-hailing travel, we counted the online car-hailing demand and the weather type in every time slice to obtain their scatter frequency map, as shown in Figure 7. Figure 7 shows that during the period from 1 to 21 January 2016, the weather in Hangzhou was mainly overcast and rainy. The average online car-hailing demand in the overcast time slice was the highest, and the demand in shower time slices was the lowest. According to Figure 7, the online car-hailing demand during rainy time slices increased with the rainfall, showing a certain positive correlation.

**Figure 8:**
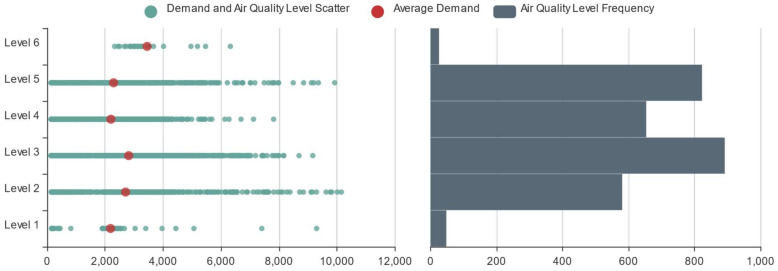
Online car-hailing demand and air quality level scatter frequency map.

To study the impacts of air quality characteristics on online car-hailing travel, we counted the online car-hailing demand and the air quality level in every time slice to obtain their scatter frequency map, as shown in Figure 8. Figure 8 shows that during the period from 1 to 21 January 2016, the air quality level of Hangzhou was above level 3, which means poor air quality. Moreover, the average online car-hailing demand during level 6 air quality time slices was the highest and that during level 1 air quality time slices was the lowest, indicating people prefer to use online car-hailing when the pollution is more serious, to avoid the harmful effects of air pollution.

**Figure 9:**
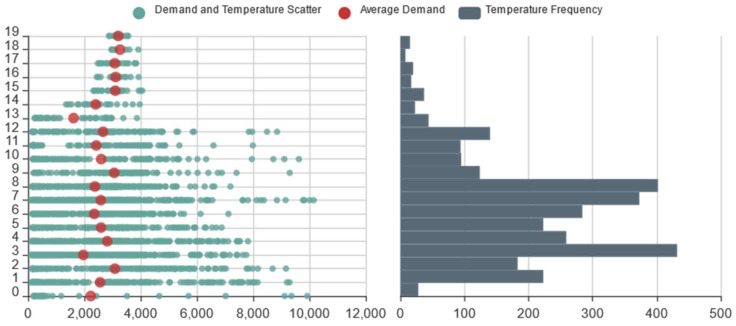
Online car-hailing demand and temperature scatter frequency map.

To study how temperature characteristics affect online car-hailing travel, we counted the online car-hailing demand and the temperature in every time slice to obtain their scatter frequency map, as shown in Figure 9. Figure 9 shows that during the period from 1 to 21 January 2016, the temperature of Hangzhou was low, mainly below 8 °C. The average online car-hailing demand during the 2 °C time slice was the highest and lowest during the 13 °C time slices. However, on the whole, we found no obvious correlation between the online car-hailing demand and temperature. We speculate that the reason for this phenomenon is the low number of days recorded in the data set, and the minimal difference in temperature in winter.

## 4. Online Car-Hailing Demand Prediction Based on a Multivariable Hybrid Time Series Model

For the time series prediction problem, due to the characteristics of linear and nonlinear time series models determine, the former can only identify the linear pattern of time series, and the advantage of the latter is that it can mine the nonlinear relationships in time series. Although the hybrid time series model based on ARIMA and LSTM solves this problem well, this kind of hybrid model is only suitable for univariate variable time series. Therefore, we used the MIC for feature selection, and fused ARIMAX and LSTM to perform data regression to construct a novel prediction model suitable for multivariate time series.

### 4.1. MIC Feature Selection

MIC feature selection is based on entropy theory. MIC feature selection has strong universality and can identify any functional relationship. It breaks through the bottleneck of the traditional feature selection method based on entropy theory only being able to deal with discrete features. Therefore, MIC feature selection can be used not only for classification problems, but also for regression problems.

For the uth feature $X_{u}=\left\{x_{u}^{i},\;i=1,\;2,\;\ldots ,\;t\right\}$ and the explained variable $Y=\left\{y_{i},\;i=1,\;2,\;\ldots ,\;t\right\}$ in data set D, the calculation process of MIC is as follows:

Step 1: Calculate the mutual information MI of $X_{u}$ and Y as:

(1) $$MI\left(X_{u},\;Y\right)=\underset{y_{i}\in Y}{\sum }\underset{x_{u}^{i}\in X_{u}}{\sum }p\left(x_{u}^{i},\;y_{i}\right)log\frac{p\left(x_{u}^{i},\;y_{i}\right)}{p\left(x_{u}^{i}\right)p\left(y_{i}\right)}$$

where $p\left(x_{u}^{i},\;y_{i}\right)$ is the joint density function of $X_{u}$ and Y, $p\left(x_{u}^{i}\right)$ is the edge probability density function of $X_{u}$, and $p\left(y_{i}\right)$ is the edge probability density function of Y.

Step 2: Divide $X_{u}$ and Y into an r∗s grid, which is recorded as $G=\left(r,\;s\right)$. To obtain the grid division that maximizes MI, normalize the value of MI to the (0,1) interval. The normalized maximum MI can be expressed as:

(2) $$MI_{D|G}\left(X_{u},\;Y\right)=\frac{MI_{D|G}^{*}\left(X_{u},\;Y\right)}{log_{min}\left\{r,\;s\right\}}$$

where $MI_{D|G}^{*}\left(X_{u},\;Y\right)$ is the maximum MI of data set D under gird G.

Step 3: Take the maximum MI under different G as the MIC; the specific calculation formula is as follows:

(3) $$\left\{\begin{array}{c} MIC\left(X_{u},\;Y\right)=\underset{r*s<B\left(n\right)}{\max}\left\{MI_{D|G}\left(X_{u},\;Y\right)\right\}\\ B\left(n\right)=n^{0.6} \end{array}\right.$$

where $B\left(n\right)$ is the maximum number of the unit grids and is a function of the samples number n.

The larger the value of $MIC\left(X_{u},\;Y\right)$, the stronger the correlation between $X_{u}$ and Y. Therefore, we calculate all the MIC values between the feature variables and the explained variable, and select features according to the following formula:

(4) $$MIC\left(X_{u},\;Y\right)\geq \sigma$$

where σ is the lowest feature selection threshold.

### 4.2. ARIMAX Linear Time Series Model

The ARIMAX model is suitable for multivariate time series modeling, as an extended ARIMA model with regression terms. The introduction of regression terms helps improve the prediction effect, and the introduced regression terms are usually the variables with a high degree of correlation with the explained variable. The ARIMA model considers that the current time series value $y_{t}$ of the stationary time series $\left\{y\right\}$ is determined by the past time series values and the external interference according to a linear expression. Therefore, the mathematical formula of ARIMA model can be written as:

(5) $$y_{t}=\delta +\overset{p}{\underset{i=1}{\sum }}\varphi_{i}y_{t-i}+\overset{q}{\underset{i=1}{\sum }}\omega_{i}\varepsilon_{t-i}$$

where $\varepsilon_{t}$ is the residual error of $\left\{y\right\}$ at time t, δ is the constant term, p is the maximum autoregressive order, $\varphi_{i}$ is the autoregressive coefficient of order i, q is the maximum moving average order, and $\omega_{i}$ is the moving average coefficient of order i.

ARIMA requires the time series to be stationary. For nonstationary time series, the d-order difference operator $\nabla^{d}y_{t}={\left(1-B\right)}^{d}y_{t}\;$ is introduced to make the time series stationary. Therefore, the final form of ARIMA is obtained as shown in Formula (6), denoted as $ARIMA\left(p,d,q\right)$.

(6) $$\nabla^{d}y_{t}=\delta +\overset{p}{\underset{i=1}{\sum }}\varphi_{i}y_{t-i}+\overset{q}{\underset{i=1}{\sum }}\omega_{i}\varepsilon_{t-i}$$

On the basis of ARIMA, ARIMAX introduces the variable sequence set $X\left(k\right)=\left\{\left\{x_{1t}\right\},\;\left\{x_{2t}\right\},\;\ldots ,\;\left\{x_{kt}\right\}\right\}$, which is highly related to the explained variable. Therefore, the final form of ARIMAX is obtained as shown in Formula (7), denoted as $ARIMAX\left(p,d,q\right)X\left(k\right)$.

(7) $$\nabla^{d}y_{t}=\delta +\overset{k}{\underset{i=1}{\sum }}\mu_{i}X_{t-i}+\overset{p}{\underset{i=1}{\sum }}\varphi_{i}y_{t-i}+\overset{q}{\underset{i=1}{\sum }}\omega_{i}\varepsilon_{t-i}$$

In the process of $ARIMAX\left(p,d,q\right)X\left(k\right)$, p,d,and q, as the input parameters of the model, need to be set in advance. The determination of parameter d is relatively simple, that is, the minimum difference order of the nonstationary sequence after processing into a stationary sequence. The Bayesian information criterion (BIC) is introduced to determine the parameters p and q. The larger the BIC value, the better the fitting effect of the model. BIC is calculated as follows:

(8) BIC=mlnn−2lnL

where m is the number of parameters, n is the number of samples, and L is the likelihood function.

Finally, some necessary statistical tests need to be conducted on the results. For the time series prediction models, they include the residual normality test and the residual autocorrelation test. The residual normality test is used to test whether the model has extracted all the useful information, only leaving unpredictable random disturbances. In this study, the Kolmogorov–Smirnov (K-S) [36] method was used for the residual normality test. The residual autocorrelation test is used to test whether there is any predictable information in the residual series. In this study, the Durbin–Watson (D-W) [37] method is used to perform the residual normality test.

### 4.3. LSTM Nonlinear Time Series Model

**Figure 10:**
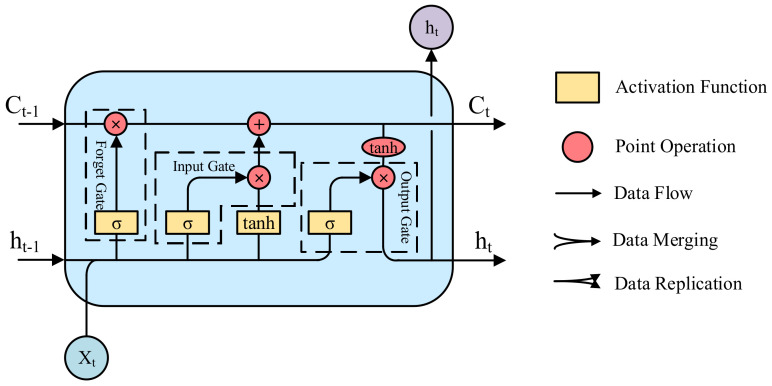
LSTM memory cell unit structure.

The LSTM network is composed of a memory cell, input gate, output gate, and forget gate. The memory cell is the basic unit of an LSTM neural network, and its specific structure is shown in Figure 10, where $X_{t}$ is the input value of the cell at time t, $C_{t}$ is the state value of the cell at time t, and $h_{t}$ is the output value of the cell at time t. The small square box with the symbol σ in the cell represents the feed-forward network layer with a sigmoid activation function. Similarly, the small square box with tanh in the cell represents the feed-forward network layer with a tanh activation function. The small round box with the *“*+*“* symbol in the cell represents a point addition operation, the small round box with *“*×*“* in the cell represents a point multiplication operation, and the small oval box with *tanh* in the cell represents the point tanh operation.

The specific operation steps of LSTM are as follows:

Step 1: Calculate the input gate value $i_{t}$ and the candidate state value $\tilde{C_{t}}$ of the cell at time t as:

(9) $$i_{t}=\delta \left(W_{i}*\left[X_{t},\;h_{t-1}\right]+b_{i}\right)$$

(10) $$\tilde{C_{t}}=tanh\left(W_{c}*\left[X_{t},\;h_{t-1}\right]+b_{c}\right)$$

where $W_{i}$ is the weight matrix of the input gate, $\left[X_{t},\;h_{t-1}\right]$ indicates connecting the vector $X_{t}$ and vector $h_{t-1}$, $b_{i}$ is the bias term of the input gate, $W_{c}$ is the weight matrix of the candidate cell state, and $b_{c}$ is the bias term of candidate cell state.

Step 2: Calculate the activation value $f_{t}$ of forget gate at time t as:

(11) $$f_{t}=\delta \left(W_{f}*\left[X_{t},\;h_{t-1}\right]+b_{f}\right)$$

where $W_{f}$ is the weight matrix of the forget gate and $b_{f}$ is the bias term of the forget gate.

Step 3: Calculate the cell state update value $C_{t}$ at time t as:

(12) $$C_{t}=i_{t}*\tilde{C_{t}}+f_{t}*C_{t+1}$$

Step 4: Calculate the output value $f_{t}$ of output gate at time t as:

(13) $$o_{t}=\delta \left(W_{o}*\left[X_{t},\;h_{t-1}\right]+b_{o}\right)$$

(14) $$h_{t}=o_{t}\tanh\left(C_{t}\right)$$

where $W_{o}$ is the weight matrix of the output gate and $b_{o}$ is the bias term of the output gate.

Through the above steps, LSTM can effectively use input value and output value to provide the long-term memory function.

### 4.4. Multivariable Hybrid Time Series Model

To improve the prediction accuracy and overcome the shortcomings of the single model, a novel multivariable hybrid time series model was constructed by combining ARIMAX and LSTM. We assumed that the complex time series was composed of a linear component and a nonlinear component. Based on the above assumption, we first applied ARIMAX model to fit the linear component of the complex time series $y_{t}$ to obtain the linear component fitting value $y_{t}^{\prime }$. Then, we determined the difference between $y_{t}$ and $y_{t}^{\prime }$ to obtain the residual error $\varepsilon_{t}$, as shown in Formula (15).

(15) $$y_{t}-y_{t}^{\prime }=\varepsilon_{t}$$

Due to the complex time series is composed of a linear and a nonlinear component, the residual error $\varepsilon_{t}$ is bound to contain the nonlinear component, which cannot be fitted by the linear model ARIMAX. Therefore, the LSTM model can be used to fit the residual error $\varepsilon_{t}$ to obtain the fitting residual error $\varepsilon_{t}^{\prime }$.

The key to the latter problem is to learn the combination method between the linear component fitting value $y_{t}^{\prime }$ and the fitting residual error $\varepsilon_{t}^{\prime }$ to obtain the final fitting value $y_{t}^{\prime \prime }$ of the complex time series $y_{t}$. Due to the complexity of the combination method, a nonlinear LSTM model with a high self-learning ability was used to learn the combination method.

(16) $$y_{t}^{\prime \prime }=f\left(y_{t}^{\prime },\;\varepsilon_{t}^{\prime }\right)$$

**Figure 11:**
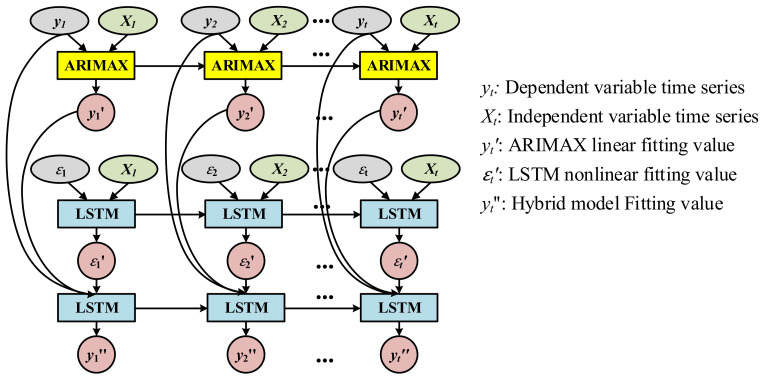
Basic structure of the multivariable hybrid time series model.

As such, the proposed multivariable hybrid time series model was constructed, and its basic structure is shown in Figure 11.

### 4.5. Verification Experiment and Result Analysis

**Figure 12:**
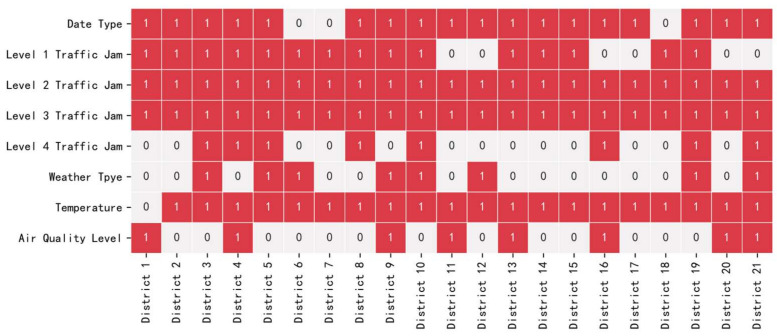
The result of MIC feature selection.

To verify the effectiveness of the proposed multivariate hybrid time series model, we conducted verification experiments on the Hangzhou online car-hailing operational data sets. Given that many variables are involved in these data sets, the MIC was firstly used to perform features section in this study. The result of MIC feature selection is shown in Figure 12, where 1 represents the variable selected and 0 represents the variable not selected.

**Figure 13:**
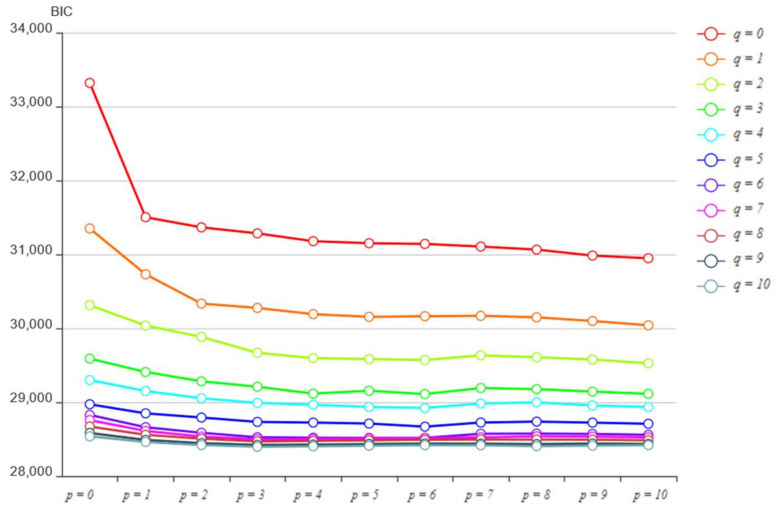
District 21 BIC value under different p and q.

Based on the result of MIC feature selection, we selected variables to construct the ARIMAX model to fit the linear component in the demand series. Firstly, the stationary of the time series was tested to determine the parameter d of ARIMAX. After the test, all the involved time series were stationary. Therefore, there was no need to determine any differences, that is, d=0. Secondly, BIC was used to determine the p and q parameters of ARIMAX. In this study, we used the ergodic method to calculate the BIC value under different p and q. Taking district 21 as an example, we calculated the BIC value under $p,\;q\in \left\{0,\;1,\;..,10\right\}$, and the specific results are shown in Figure 13. Figure 13 shows that with the increase in p and q, BIC decreases gradually, but the decrease amplitude is increasingly smaller, and finally tends to be stable. As increases in p and q lead to a large increase in computational complexity, p and q with a relatively low BIC value can be selected as input parameters of ARIMAX. After the parameters of ARIMAX are determined, the linear component in the demand series can be fitted to obtain the linear component fitting value $y_{t}^{\prime }$ and the residual error $\varepsilon_{t}$. Finally, we conducted the K-S normality test and the D-W normality test on the residual series.

Next, we used LSTM to fit the residual error $\varepsilon_{t}$ to obtain the fitting residual error $\varepsilon_{t}^{\prime }$. Finally, we used LSTM to learn the combination method between the linear component fitting value $y_{t}^{\prime }$ and the fitting residual error $\varepsilon_{t}^{\prime }$ to obtain the final fitting value $y_{t}^{\prime \prime }$. The parameters set for the LSTM model are shown in Table 5.

**Figure 14:**
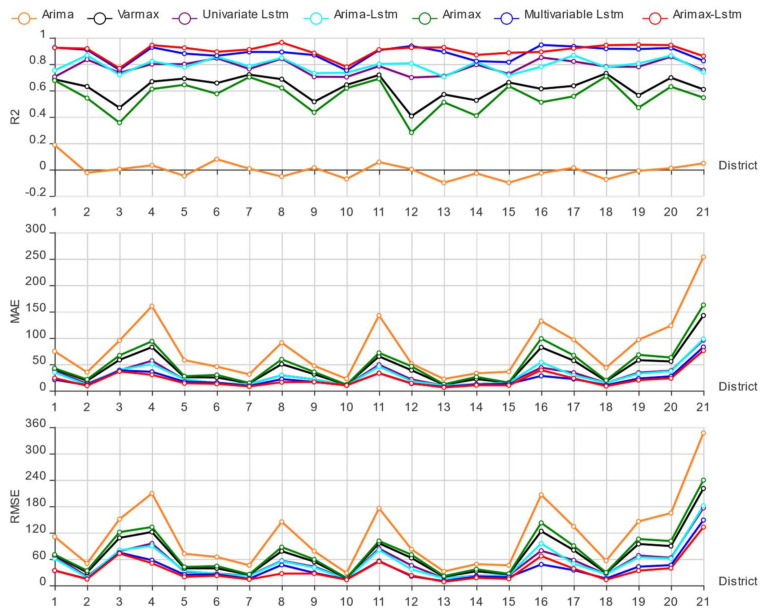
Performance indicators of six prediction models.

Finally, the performance of the trained multivariate hybrid time series model was evaluated on the test sets. To verify the superiority of the proposed model, we set up the control groups: the ARIMA model, vector autoregressive moving average model with exogenous regressors (VARMAX) model, univariate LSTM model, ARIMA-LSTM model, ARIMAX model, and multivariate LSTM model. To more accurately evaluate the performance of each model, goodness of fit $\left(R^{2}\right)$, mean absolute error (MAE), and root mean square error (RMSE) were used to evaluate the six prediction models in this study. Among them, the larger the $R^{2}$, the better model fitting effect; the smaller the MAE, the lower the model error; the smaller the RMSE, the lower model volatility. The specific evaluation results are shown in Figure 14.

Firstly, by examining Figure 14 horizontally, it can be found that different models on different districts data sets perform differently. For example, on the district 9 and 19 data sets, the nonlinear models generally performed better, whereas the linear generally showed worse performance; on the district 1 and 10 data sets, the linear models generally performed better, and that of the nonlinear model was generally lower. We speculate that this may be caused by the different proportions of linear and nonlinear components in the data set. The fitting effect of the linear models on the data sets with a higher linear component is better, whereas that of the nonlinear models in the data sets with a higher nonlinear component is better. Figure 14 also shows that the index curves of different models display almost the same change trend in some districts. For example, the performance indicators of almost all models are high on the district 4, 11, and 20 data sets, whereas the performance indicators of almost all models are low on the district 3, 9, and 12 data sets. We speculated that this may be caused by the nature of the data sets, such as volatility and stability.

Then, examining Figure 14 vertically, compared with the other prediction models, the proposed ARIMAX-LSTM model has the largest $R^{2}$ and the smallest MAE and RMSE in most districts test sets, so the fitting effect of the model is the best and the error is the smallest. Therefore, the ARIMAX-LSTM model proposed in this study provides improved prediction performance. For all single models, Figure 14 shows that the ARIMA, VARMAX, and ARIMAX models, as three linear time series models, performed the worst, which means the LSTM chain improves performance. However, the ARIMAX model with explanatory variables performed much better than the ARIMA model without explanatory variables, which means the performance of prediction model can be improved by adding explanatory variables. The VARMAX model with exogenous variables performed better than the ARIMAX model without exogenous variables, which means the performance of a prediction model can be improved by adding exogenous variables. The same conclusion can also be drawn by comparing the performance of the univariate and multivariate LSTM models or comparing the performance of the ARIMA-LSTM model with ARIMAX-LSTM model. From Figure 14, it can be seen that the performance of the ARIMA-LSTM model is better than that of the ARIMA model and univariate LSTM model, and the performance of the ARIMAX-LSTM model is better than that of the ARIMAX model and multivariate LSTM model. Therefore, we concluded that the performance of the hybrid model is better than that of the single models.

## 5. Conclusions

We aimed to fill a research gap by analyzing the characteristics of online car-hailing and by overcoming the shortcomings of univariate hybrid time series models in traffic prediction. We studied online car-hailing travel characteristics from multiple dimensions such as district, time, traffic jams, weather, air quality, and temperature based on the online car-hailing operational data sets. We also proposed a novel traffic prediction model suitable for multivariate time series based on MIC, ARIMAX, and LSTM, and verified the effectiveness of the proposed model on the online car-hailing operational data sets. Finally, we drew the following conclusions:

(1) In these districts with the larger online car-hailing demand, the passenger inflow and outflow is also larger, but it is more difficult to find a taxi.

(2) Daily online car-hailing travel usually presents obvious double hump feature, that is, the morning rush and the evening rush peaks are obvious. However, differences exist on in nonworking day and in different districts.

(3) From 1 to 21 January 2016, the online car-hailing demand in Hangzhou showed a significant positive correlation with traffic jams.

(4) Due to the fewer days recorded in the data sets, we found no obvious correlation between online car-hailing demand and weather type, air quality, or temperature in Hangzhou from 1 to 21 January 2016.

(5) The performance of the multivariable hybrid time series ARIMAX-LSTM model proposed in this paper is better than that of univariate the hybrid time series ARIMA-LSTM model.

(6) Univariate LSTM, ARIMA-LSTM, multivariate LSTM, and ARIMAX-LSTM, as linear time series models, perform better than the nonlinear time series models ARIMA and ARIMAX.

(7) The addition of explanatory variables and exogenous variables can improve the performance of time series models.

## 6. Discussion

In this study, we analyzed the online car-hailing travel characteristics from multiple dimensions and proposed a novel multivariable hybrid time series traffic prediction model based on online car-hailing operational data sets. Compared with the existing research, our research provides the following innovations:

(1) A set of travel characteristics was analyzed and a traffic prediction modeling method for online car-hailing was constructed, closing the related research gap.

(2) We systematically and comprehensively analyzed the online car-hailing travel characteristics from multiple dimensions. In addition to the common dimensions of district, time, weather, air quality, and temperature, we also studied the impact of traffic jams.

(3) We proposed a novel traffic prediction model suitable for multivariate time series based on MIC, ARIMAX, and LSTM. Compared with the traditional univariate hybrid time series model ARIMA-LSTM, the performance of the proposed model is significantly improved by adding explanatory variables.

In summary, we analyzed the online car-hailing travel characteristics from multiple dimensions, identifying some meaningful online car-hailing travel characteristics, and proposed a novel multivariate time series traffic prediction model to improve the prediction accuracy. This analysis of this set of travel characteristics and the traffic prediction modeling method provides a new idea for the ride-hailing industry. Based on our research, we suggest that the ride-hailing industry actively apply sensor and IoT technologies to establish big data travel platforms to support travel characteristics mining and travel prediction modeling. Then, these mined travel characteristics and predicted demand should be considered in the creation of an order distribution strategy to further improve operational efficiency. In the future, we will actively seek new cooperation to obtain more online car-hailing operation data sets, analyze the online car-hailing travel characteristics from broader and deeper dimensions, and try to develop a new hybrid time series model based only on the nonlinear time series models.

## Figures and Tables

**Table 1:** Order data set fields description.

Field Name	Description	Example
order_id	Order ID	0e0d61fe14b76b59a83c421a720216a5
driver_id	Driver ID	f214b0789124b60ea8e279543da45c78 or Null
passenger_id	Passenger ID	a083fd0a2181a13d7a614271edd4a0af
start_district_id	Order start district ID	74c1c25f4b283fa74a5514307b0d0278
dest_district_id	Order destination district ID	dd8d3b9665536d6e05b29c2648c0e69a
price	Order price	10.7
datetime	Order date and time	2016-01-17 20:15:26

**Table 2:** Traffic jam data set fields description.

Field Name	Description	Example
district_id	District ID	1ecbb52d73c522f184a6fc53128b1ea1
traffic	Road quantity in different traffic jam levels	1:231 2:33 3:13 4:10
datetime	Records the date and time	2016-01-01 23:30:22

**Table 3:** Weather data set fields description.

Field Name	Description	Example
datetime	Record date and time	1 January 2016, 09:55:15
weather	Weather type	2
temperature	Temperature (°C)	4.0
air_quality	Air quality level	3

**Table 4:** District 16 combined demand data.

District ID	Date	Time Slice ID	Demand	Traffic Jam Level 3	Temperature	Air Quality Level
16	1 January 2016	1	101	76	3	4
16	1 January 2016	2	116	76	3	4
16	2016-01-01	3	113	86	3	4
16	21 January 2016	142	65	70	1	1
16	21 January 2016	143	64	75	1	1
16	21 January 2016	144	52	70	1	1

**Table 5:** The parameters setting of LSTM model.

Parameter	Value
Time Steps	6
Input Layer Units Number	47
Output Layer Units Number	1
Hide Layer Number	1
Hide Layer Units Number	100
Epochs	60
Batch Size	16
Activation Function	Rectified linear unit (ReLU)
Loss Function	Min mean absolute error (MAE)
Optimizer	Adam
Dropout	0.5

## Data Availability

Not applicable.
